# Sex-Based Differences in BMI and LDL-C Trajectories Following Type 1 Diabetes Diagnosis in Youth

**DOI:** 10.1155/pedi/7996152

**Published:** 2025-11-11

**Authors:** Emir Tas, Swetha Movva, Uma Muzumdar, Ingrid Libman

**Affiliations:** ^1^Department of Endocrinology and Diabetes, UPMC Children's Hospital of Pittsburgh, Pittsburgh, Pennsylvania, USA; ^2^Department of Pediatrics, University of Pittsburgh School of Medicine, Pittsburgh, Pennsylvania, USA

**Keywords:** BMI trajectory, cardiometabolic risk, LDL-C, type 1 diabetes, youth

## Abstract

**Introduction:**

Cardiovascular disease (CVD) disproportionately affects females with type 1 diabetes (T1D), yet the emergence of sex-specific metabolic risk during early disease remains unclear. We evaluated whether sex differences in BMI percentile (BMIp) and LDL-cholesterol (LDL-C) trajectories appear within the first 2 years following T1D diagnosis.

**Methods:**

We conducted a retrospective cohort study of 542 youth with new-onset T1D (mean age 10.4 ± 3.9 years; 54.1% male) and assessed sex differences in BMIp and LDL-C trajectories using linear mixed-effects models, adjusting for age, HBA1c and diabetic ketoacidosis (DKA) status at diagnosis, and baseline weight category (LDL-C model only).

**Results:**

At diagnosis, median BMIp did not differ by sex (females: 50.9 [IQR: 19.2–84.1] vs. males: 63.0 [17.8–93.0]; *p*=0.15). Over 2 years, females experienced significantly greater BMIp increases (median change: 27.4 [5.1, 49.7] vs. 13.1 [–4.3, 30.5] percentage points; *p*=0.002). Adjusted models confirmed steeper increases in BMIp for females compared to males (sex × time interaction: 7.54 [3.13, 11.94]; *p*  < 0.001). LDL-C was higher in females at diagnosis (2.51 ± 0.80 vs. 2.30 ± 0.70 mmol/L [97 ± 31 vs. 89 ± 27 mg/dL]; *p*=0.003) and follow-up (2.25 ± 0.59 vs. 2.12 ± 0.65 mmol/L [87 ± 23 vs., 82 ± 25 mg/dL]; *p*=0.02), with adjusted models confirming a persistent difference (0.17 [0.06, 0.27] mmol/L [6.39 [2.39, 10.40] mg/dL]; *p*=0.002).

**Discussion:**

Females with T1D exhibit steeper early increases in adiposity and persistently higher LDL-C levels compared to males, independent of age, glycemic control, and DKA status at diagnosis. These findings underscore the importance of sex-specific metabolic monitoring and early intervention beginning at diagnosis to mitigate long-term cardiovascular risk.

## 1. Introduction

Cardiovascular disease (CVD) is a leading cause of morbidity and mortality in individuals with type 1 diabetes (T1D), a condition affecting nearly 2 million people in the United States [1, 2]. Among those diagnosed in childhood, CVD risk emerges early and persists into adulthood, with both sexes facing significantly higher risk than the general population [3]. Strikingly, adult females with T1D experience nearly twice the risk of CVD events compared to males—a reversal of the cardioprotective effect typically seen in nondiabetic populations [4, 5]. Although sex differences in metabolic risk factors—such as higher BMI, adverse lipid profiles, and greater insulin resistance—are well documented in adolescents with T1D [6], the timing and early evolution of these disparities, particularly in the period following diagnosis, remain poorly understood.

The first 2 years after T1D onset—including the partial clinical remission (“honeymoon”) phase—represent a unique metabolic window. During this phase, residual endogenous insulin production and improved glycemic control may temporarily mask early metabolic abnormalities [7]. Yet few studies have evaluated sex-specific trajectories of key cardiovascular risk factors, such as BMI percentile (BMIp) and LDL-cholesterol (LDL-C), beginning at the time of diagnosis. As a result, it is unclear when these differences first arise and whether early divergence contributes to long-term cardiovascular risk.

This question is particularly important given the “metabolic memory” hypothesis, which suggests that early exposure to dyslipidemia and hyperglycemia can trigger lasting molecular changes that increase future complication risk—even after glycemic control improves [7]. Because atherosclerosis often begins silently in childhood, early identification of cardiometabolic risk is especially important in youth with T1D [8]. Supporting this, the DCCT/EDIC trial demonstrated that early intensive therapy reduces long-term CVD risk [9], and hormonal changes during puberty—especially in females—may further exacerbate insulin resistance and lipid abnormalities [10]. Identifying when sex differences in these risk factors emerge may help define critical windows for prevention.

In this study, we examined sex differences in BMIp and LDL-C trajectories over the first 2 years following T1D diagnosis in youth. We hypothesized that females would exhibit greater increases in BMIp and persistently higher LDL-C levels than males during this early period, independent of baseline HbA1c, age at diagnosis, and other clinical factors. These findings aim to inform earlier, sex-specific strategies for cardiovascular risk reduction in this high-risk population.

## 2. Methods

### 2.1. Study Population

We conducted a retrospective cohort study of pediatric and adolescent patients aged 2 to <18 years with newly diagnosed T1D who were admitted to UPMC Children's Hospital of Pittsburgh between January 1, 2019 and December 31, 2021. At our institution, all patients with new-onset T1D are routinely hospitalized for initial diabetes education and insulin dose titration. Patients were identified through electronic health record (EHR) review, yielding an initial cohort of 769 individuals.

Inclusion criteria were hospitalization for new-onset diabetes, initiation of insulin therapy, and a confirmed diagnosis of T1D based on provider assessment, laboratory findings (e.g., positive diabetes autoantibodies if obtained), and clinical documentation. We excluded individuals who were <2 or ≥18 years of age at diagnosis (*n* = 39), diagnosed with type 2 diabetes (*n* = 129), or had other forms of diabetes, including medication-induced or cystic fibrosis-related diabetes (*n* = 35). An additional 24 patients were excluded due to missing fasting lipid profile data at admission.

**Figure 1:**
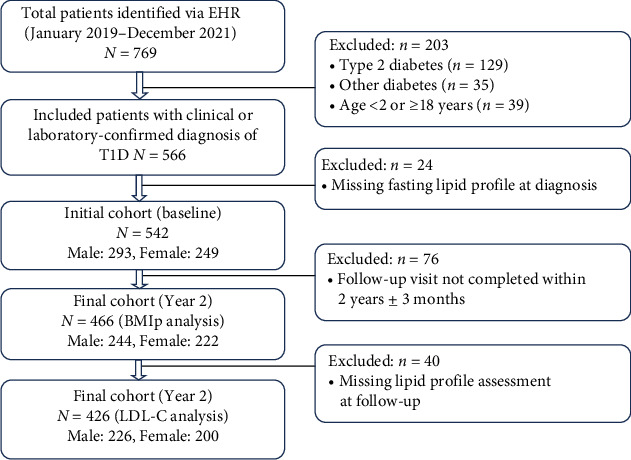
Flowchart of the study participants.

After applying these criteria, the final analytic sample included 542 patients (293 males and 249 females) with baseline data. Follow-up data at 2 years (±3 months) postdiagnosis were available for 466 patients (244 males and 222 females) (Figure 1).

### 2.2. Data Collection

Demographic and clinical data were obtained through retrospective chart review. Collected variables included age at diagnosis, sex, self-reported race, weight, height, BMI, and BMIp for age and sex, as well as initial venous blood gas, serum bicarbonate, and HbA1c. Weight, BMI, and BMIp were derived from the first recorded measurements upon arrival to our institution. Diabetic ketoacidosis (DKA) at diagnosis was defined using ISPAD 2022 criteria as venous pH <7.30 or serum bicarbonate <18 mmol/L [11].

Weight categories were determined based on BMIp for age and sex according to CDC definitions: <3rd percentile as underweight, 3rd–<85th percentile as normal weight, 85th–<95th percentile as overweight, and ≥95th percentile as obese.

Fasting lipid profiles—including LDL-C, HDL-C, total cholesterol, and triglycerides—were obtained prior to discharge, typically 2–3 days after admission, once ketosis had resolved and insulin therapy was stabilized. Follow-up lipid and anthropometric measurements were obtained from outpatient visits closest to 2 years postdiagnosis (±3 months). LDL-C was measured using a direct assay, minimizing the impact of fasting status. Basal insulin dose (units/kg/day) was recorded at both time points; total daily dose was not analyzed due to inconsistent documentation across charts. All laboratory values are reported in SI units, with conventional units provided in parentheses where appropriate.

All participants were initiated on multiple daily injections (MDIs) during hospitalization for new-onset T1D. Per institutional policy, transition to insulin pump therapy required completion of structured pump education classes and typically occurred ≥3 months after diagnosis. Treatment modality at year 2 (MDI vs. pump) was recorded and examined by sex.

In the primary analysis, age at diagnosis was included as a continuous covariate to reflect developmental stage across the cohort. In exploratory sensitivity analyses, pubertal status was approximated using three different age-based cutoffs [1]: females <9 years and males <10 years [2]; females <9 years and males <11 years; and [3] females <10 years and males <11 years, each categorized as prepubertal [11, 12]. Tanner staging was inconsistently documented at diagnosis and was therefore not included in the analysis. These exploratory models were conducted to assess the robustness of observed sex differences, given that pubertal onset is associated with physiological increases in insulin resistance and changes in adiposity and lipid profiles [13].

### 2.3. Statistical Analysis

Continuous variables were summarized as means and standard deviations or medians with interquartile ranges, while categorical variables were presented as frequencies and percentages. Baseline comparisons between male and female participants were conducted using independent samples *t*-tests or nonparametric equivalents for continuous variables, and chi-square tests for categorical variables. A two-sided *p*-value <0.05 was considered statistically significant. All analyses were performed using IBM SPSS Statistics (version 29).

The primary outcomes were BMIp and LDL-C, each assessed at diagnosis and again at 2 years postdiagnosis (±3 months). Longitudinal changes and sex differences were evaluated using linear mixed-effects regression models with random intercepts to account for within-subject correlations. Fixed effects included time (diagnosis vs. follow-up), sex, the sex × time interaction, and covariates: age at diagnosis (modeled as a continuous variable), baseline HbA1c, DKA status at diagnosis, and baseline weight category (included only in the LDL-C model). The sex × time interaction tested whether the change in BMIp or LDL-C over time differed between males and females. Degrees of freedom for fixed effects were estimated using the Satterthwaite approximation to account for unequal group sizes and the hierarchical structure of the data. HbA1c is reported in both mmol/mol and percent, as needed, and percent was used in the modeling tables.

In exploratory sensitivity analyses, age at diagnosis was replaced with categorical pubertal stage variables using three different age-based definitions to evaluate robustness. In all models, females below the designated age threshold were classified as prepubertal, as were males below the corresponding male cutoff. The definitions tested were [1]: females <9 years and males <10 years [2]; females <9 years and males <11 years; and [3] females <10 years and males <11 years. Each model retained the same structure as the primary analysis, including time, sex, and the sex × time interaction as fixed effects, while omitting higher-order interaction terms involving pubertal status. These sensitivity analyses were conducted to assess whether the observed sex differences in metabolic trajectories were consistent across varying definitions of prepubertal status.

Model assumptions were evaluated using residual diagnostics. A complete case approach was used, assuming data were missing at random. Of the 542 patients at baseline, follow-up data were available for 466 (85.9%) for BMIp and 426 (78.6%) for LDL-C. For LDL-C, there were no significant differences in baseline characteristics—including sex, HbA1c, BMIp, LDL-C, weight category, or DKA status—between those with and without follow-up data (all *p*  > 0.1), supporting the plausibility of the missing-at-random assumption. For BMIp, small but statistically significant differences in sex and weight category were observed between those with and without follow-up (*p*=0.042 and *p*=0.040, respectively), which may reflect modest sampling bias (Tables S1 and S2).

### 2.4. Ethical Approval

This study was approved by the University of Pittsburgh Institutional Review Board and deemed exempt from the requirement for informed consent (IRB number STUDY23080180).

## 3. Result

### 3.1. Characteristics of the Study Participants at Diagnosis and 2-Year Follow-Up

The mean age at diagnosis for the full cohort (*n* = 542) was 10.4 ± 3.9 years, with a mean HbA1c of 109 ± 28 mmol/mol (12.1 ± 2.6%). The sample was 54.1% male and 45.9% female, and most participants (91%) identified as White. At diagnosis, 48.5% of the subjects presented with DKA. The median BMIp for the entire cohort at diagnosis was 56.2% (IQR: 20.5–89.3). Based on CDC-defined BMIp categories, 11.4% were underweight, 58.3% were of normal weight, 11.3% were overweight, and 19.0% were obese.

**Figure 2:**
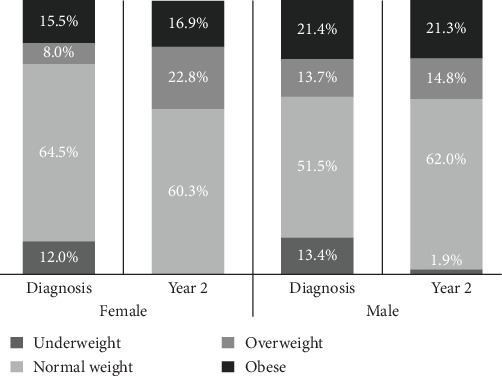
Sex differences in weight status at diagnosis and 2 years postdiagnosis in youth with T1D. Bars represent the distribution of weight categories (underweight, normal weight, overweight, and obese) at diagnosis and at 2-year follow-up, stratified by sex. Females showed a decline in the proportion of normal weight status and an increase in overweight prevalence, while males demonstrated a modest increase in normal weight and overweight categories. Underweight rates decreased over time in both sexes.

Clinical and laboratory characteristics stratified by sex at diagnosis and follow-up are presented in Table 1. At diagnosis, females were significantly younger than males (9.9 ± 3.9 vs. 10.9 ± 3.9 years, *p*=0.002), and had higher HbA1c levels (113 ± 30 mmol/mol [12.5 ± 2.7%] vs. 104 ± 27 mmol/mol [11.7 ± 2.5%], *p*  < 0.001), LDL-C (2.50 ± 0.80 mmol/L [96.7 ± 30.8 mg/dL] vs. 2.31 ± 0.69 mmol/L [89.2 ± 26.6 mg/dL], *p*=0.003), and total cholesterol (4.06 ± 1.02 mmol/L [157.1 ± 39.5 mg/dL] vs. 3.86 ± 0.84 mmol/L [149.1 ± 32.5 mg/dL], *p*=0.011). Median BMIp at diagnosis did not differ significantly between sexes (50.9 [19.2, 84.1] for females vs. 63.0 [17.8, 93.0] for males, *p*=0.149). However, at diagnosis, weight category distribution differed by sex (*p*=0.013). A greater proportion of females were in the normal weight range (64.5% vs. 51.5%), whereas more males were classified as overweight (13.7% vs. 8.0%) or obese (21.4% vs. 15.5%). The proportion of underweight participants was similar (12.0% for females vs. 13.4% for males). By the 2-year follow-up, sex-based differences in weight category distribution persisted (*p*=0.028), but the pattern had shifted. Females, who were initially less likely to be overweight, became more likely than males to fall into the overweight category (22.8% vs. 14.8%). Although females remained less likely to be obese, the gap narrowed (16.9% vs. 21.3%), reflecting a relative increase in obesity among males and a notable upward shift in weight status among females over time (Figure 2).

There were no significant differences in HbA1c between females and males (62 ± 19 mmol/mol [7.8 ± 1.7%] vs. 61 ± 18 mmol/mol [7.7 ± 1.6%], *p*=0.49) at the 2-year follow-up. However, females continued to exhibit significantly higher total cholesterol (4.22 ± 0.80 mmol/L [163 ± 31 mg/dL] vs. 4.01 ± 0.80 mmol/L [155 ± 31 mg/dL], *p*=0.01) and LDL-C levels (2.25 ± 0.59 mmol/L [87 ± 23 mg/dL] vs. 2.12 ± 0.65 mmol/L [82 ± 25 mg/dL], *p*=0.02). Median BMIp remained similar between sexes at follow-up (females: 78.3 [56.0, 93.5] vs. males: 76.1 [46.8, 93.8], *p*=0.24), despite females showing significantly greater increases in BMIp from diagnosis (median change: 27.4 [5.1, 49.7] vs. 13.1 [–4.3, 30.5] percentage points, *p*=0.002). Basal insulin requirements (units/kg/day) did not differ by sex at either baseline (*p*=0.59) or follow-up (*p*=0.22) (Table 1). At year 2, 210 of 466 participants (45.0%) had transitioned to insulin pump therapy. Pump uptake did not differ significantly by sex (females: 49.1% vs. males: 41.4%; *p*=0.115).

### 3.2. BMIp Trajectories

**Figure 3:**
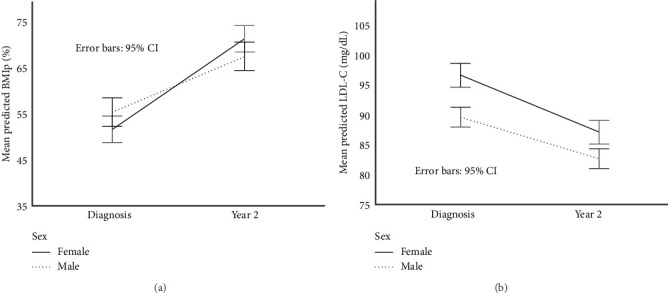
Sex-based trajectories of BMI percentile (A) and LDL-C (B) over the first 2 years following T1D diagnosis. Panel A shows changes in BMI percentile from diagnosis to 2-year follow-up, stratified by sex. Panel B displays LDL-C levels over the same period, also stratified by sex. Values represent group means with 95% confidence intervals. A significant sex × time interaction was observed for BMI percentile (*p*  < 0.001), indicating greater increases in females. LDL-C remained significantly higher in females at both timepoints, although the pattern of change over time was similar between sexes (*p*=0.307 for interaction). LDL-C is presented in conventional units (mg/dL) for interpretability. SI units (mmol/L) are used elsewhere in the manuscript.

In the primary linear mixed-effects model using the long-format dataset, BMIp increased significantly from diagnosis to follow-up (*β* = –19.90 percentage points, 95% CI: –23.08 to –16.77, *p*  < 0.001). Females experienced a significantly greater increase in BMIp over time compared to males, as indicated by a significant sex × time interaction (*β* = 7.71 percentage points, 95% CI: 3.34–12.07, *p*  < 0.001). At the 2-year follow-up, BMIp remained significantly higher in females than males (*β* = –5.40 percentage points, 95% CI: –10.34 to –0.46, *p*=0.032), after adjusting for covariates (Figure 3A).

Older age at diagnosis was significantly associated with higher BMIp across the two timepoints (*β* = 1.05 percentage points per year, 95% CI: 0.41–1.69, *p*=0.001). In contrast, baseline HbA1c (*p*=0.471) and DKA status at diagnosis (*p*=0.347) were not significantly associated with BMIp (Table 2). These results reflect average associations with BMIp across the study period, rather than associations with change over time or values at a specific timepoint.

In sensitivity analyses, we evaluated the impact of different age-based definitions of pubertal status on BMIp trajectories. Three models were tested [1]: female <9 and male <10 years as prepubertal [2]; female <9 and male <11 years; and [3] female <10 and male <11 years. Across all definitions, pubertal participants demonstrated significantly higher BMIp compared to their prepubertal peers (*β* range: 6.67–8.29, all *p*  < 0.01). Importantly, the sex × time interaction remained robust and statistically significant in all models (*β* ≈ 7.7, *p*  < 0.001), confirming that females experienced greater BMIp gains over time than males, independent of pubertal classification. These findings support the consistency of observed sex differences in early BMIp trajectories following T1D diagnosis (Table S3).

In sensitivity analyses using BMI *z*-scores (BMIzs), females again showed greater increases over time compared with males (sex × time *β* = –0.32, 95% CI –0.64 to 0.01, *p*=0.055; Table S4). Although this narrowly missed statistical significance, the direction and magnitude were consistent with our BMIp models.

### 3.3. LDL-C Trajectories

In the final linear mixed-effects model, LDL-C levels were significantly higher at diagnosis than at the 2-year follow-up (*β* = 0.25 mmol/L [9.61 mg/dL], 95% CI: 0.15, 0.34 mmol/L [5.92, 13.30 mg/dL], *p*  < 0.001). Males had lower LDL-C levels than females across timepoints (*β* = –0.13 mmol/L [–5.00 mg/dL], 95% CI: –0.25, –0.01 mmol/L [–9.55, –0.44 mg/dL], *p*=0.032), but the sex × time interaction was not significant (*p*=0.307), indicating that the pattern of LDL-C change over time was similar for both sexes (Figure 3B).

Several clinical variables were associated with LDL-C levels across the two timepoints. Older age at diagnosis (β = 0.03 mmol/L [1.04 mg/dL] per year, 95% CI: 0.01, 0.04 mmol/L [0.52, 1.56 mg/dL], *p*  < 0.001) and higher HbA1c at diagnosis (β = 0.04 mmol/L [1.66 mg/dL] per 11 mmol/mol [1%] increase, 95% CI: 0.02, 0.06 mmol/L [0.86, 2.46 mg/dL], *p* < 0.001) were independently associated with higher LDL-C levels overall. Participants classified as overweight or obese at diagnosis also had higher LDL-C values compared to those with normal weight (*β* = 0.07 mmol/L [2.54 mg/dL], 95% CI: 0.01, 0.12 mmol/L [0.37, 4.72 mg/dL], *p*=0.022). DKA status at diagnosis was not significantly associated with LDL-C levels (*p*=0.725) (Table 3).

In exploratory analyses, we assessed the effect of alternative age-based definitions of pubertal status on LDL-C levels. Three classification strategies were evaluated [1]: females <9 and males <10 years [2]; females <9 and males <11 years; and [3] females <10 and males <11 years considered prepubertal. Across all definitions, pubertal participants demonstrated significantly higher LDL-C compared to their prepubertal peers (*β* range: 0.17–0.18 mmol/L [6.50–6.84 mg/dL], all *p*  < 0.01). The association between higher baseline weight category and elevated LDL-C remained significant across models. These findings support the robustness of the observed pubertal effect on LDL-C, independent of other covariates (Table S5).

## 4. Discussion

Our study demonstrates that sex differences in cardiometabolic risk factors—namely BMIp and LDL-C—emerge within the first 2 years following T1D diagnosis. Females exhibited greater increases in BMIp over time and had higher LDL-C levels at both diagnosis and follow-up, compared to males. These findings were observed despite similar baseline BMIp and after adjusting for age, HbA1c, and DKA status.

The significant sex × time interaction in BMIp suggests a steeper adiposity trajectory in females during early disease. Although the adjusted BMIp difference at follow-up was modest, the consistent divergence over time may hold clinical relevance. LDL-C levels remained higher in females across timepoints, consistent with prior studies in healthy adolescents [14], though large datasets like NHANES report less consistent sex differences, possibly due to age or pubertal variability [15]. Our findings suggest that in the context of T1D, sex-based metabolic divergence may emerge earlier than previously recognized and may be independent of glycemic control and pubertal status.

The observed early sex differences in BMIp and LDL-C trajectories likely reflect a combination of biological and behavioral influences. Prior research has demonstrated that females exhibit lower insulin sensitivity than males, even in healthy pediatric populations, and this imbalance may be further exacerbated in the setting of T1D [10, 16]. In addition, sex hormones—particularly estrogen—play a critical role in lipid regulation and fat distribution, with effects that may precede visible pubertal changes [17]. Our age-stratified sensitivity analyses reinforce the possibility that such mechanisms may begin influencing cardiometabolic profiles before overt puberty.

Behavioral factors, including dietary intake, physical activity patterns, and psychosocial stress responses, may also contribute to these early disparities [18, 19]. Although prior studies have suggested that females may receive higher insulin doses postdiagnosis [20], our data did not show significant sex differences in basal insulin requirements at diagnosis or follow-up. However, we were unable to evaluate total daily insulin dose due to inconsistent charting, limiting our ability to fully assess dosing differences. These findings suggest that the early divergence in metabolic risk is unlikely to be solely attributable to insulin exposure.

Our findings align with prior work by Brown et al. [21], who reported worse cardiometabolic profiles in adolescent females with long-standing T1D (mean 8.6 ± 3.1 years)—including higher LDL-C, BMIzs, and inflammatory markers—independent of glycemic control or adiposity. While those differences were attributed in part to pubertal hormonal changes, our study extends these observations by showing that metabolic divergence begins early, during the first 2 years after diagnosis, even within the partial clinical remission phase. Together, these studies reinforce the need for earlier, sex-specific risk identification and monitoring.

Current U.S. guidelines from the American Diabetes Association and the American Heart Association recommend initiating statin therapy in youth with T1D based on overall cardiovascular risk, rather than fixed LDL-C thresholds alone. Statins are generally considered when LDL-C is ≥4.14 mmol/L (≥160 mg/dL) in youth without additional risk factors, and at lower thresholds (≥3.36 mmol/L [≥130 mg/dL] or even ≥2.59 mmol/L [≥100 mg/dL]) when additional risk factors—such as obesity, hypertension, or a strong family history—are present [22, 23]. In contrast, European guidelines, including those from the European Atherosclerosis Society, adopt more aggressive thresholds, recommending treatment when LDL-C exceeds 1.81 mmol/L (70 mg/dL) after 10 years of diabetes duration, or 1.42 mmol/L (55 mg/dL) after 20 years [24]. These lower thresholds are based on modeling studies and expert consensus suggesting that cumulative LDL-C exposure—also known as “cholesterol-years”—is a key driver of atherosclerosis, and that earlier intervention may confer greater long-term cardiovascular protection in high-risk populations such as youth with T1D [25]. Our findings raise the possibility that some youth—particularly females with early increases in BMIp—may benefit from closer monitoring and individualized risk assessment. While our study does not assess clinical outcomes or effects of intervention, these early patterns support the rationale for considering sex-specific stratification and for exploring the timing and intensity of preventive strategies in future studies.

As antiobesity medications (AOMs) become more widely available in pediatrics, females with T1D and early weight gain may represent a particularly high-risk subgroup for future intervention. Glucagon-like peptide-1 receptor agonists (GLP-1 RAs) have shown benefit in adolescents with obesity and type 2 diabetes, improving both weight and lipid profiles [26, 27]. However, their use in youth with T1D remains largely unexplored, and existing studies are limited [28]. Given the complex relationship between insulin therapy, adiposity, and cardiovascular risk in T1D, AOMs may offer a promising adjunctive strategy—particularly if introduced early, during or shortly after the honeymoon phase, when metabolic trajectories may still be modifiable. Although our study does not assess treatment effects, the observed early divergence in BMIp may highlight a potential window for future intervention studies. Pharmacologic agents, such as GLP-1 RAs, warrant further investigation to determine their safety and efficacy in this population. Prospective trials are needed to determine both the safety and long-term cardiometabolic benefits of such therapies in this unique population.

While the absolute LDL-C differences observed in our cohort were modest, they may still carry clinical significance. For example, adult studies suggest that every 0.26 mmol/L (10 mg/dL) reduction in LDL-C is associated with a 5%–6% relative reduction in major cardiovascular events [29, 30]. Therefore, approximately 0.16 mmol/L (6 mg/dL) higher LDL-C observed in females in our cohort may represent a meaningful early risk marker, particularly in a population already predisposed to additional risk burden for cardiovascular morbidity. These findings support the need for earlier, sex-specific lipid screening and consideration of tailored intervention thresholds. Additionally, given that current ISPAD guidelines recommend lipid screening starting at age 11 and repeating every 3 years if normal [31], our results suggest that more frequent or earlier screening may be warranted in females—especially those with early BMIp increases. This may help identify youth at elevated cardiovascular risk even before traditional treatment thresholds are met.

This study has several limitations. First, we used age-based cutoffs as a proxy for pubertal status due to inconsistent documentation of Tanner staging. However, we addressed this limitation by conducting sensitivity analyses using multiple age-based definitions. Second, as a retrospective study, our analysis is subject to potential confounding from unmeasured variables, such as dietary habits, physical activity, psychosocial stress, or genetic factors. Third, the study was conducted at a single tertiary pediatric center with a predominantly nonHispanic White population, which may limit generalizability. Although prehospital weight data were not available, we used the earliest recorded anthropometrics and obtained reliable laboratory data at diagnosis. However, these values may underestimate true anthropometric status due to dehydration commonly present at diagnosis. A small degree of attrition bias is possible, as participants with missing follow-up BMIp data differed slightly in baseline sex and weight category distributions. However, given the large sample size, consistent patterns across models, and robust sensitivity analyses, we believe the impact on overall findings is minimal. Although treatment regimen may influence weight and lipid outcomes, all patients in our cohort were initiated on MDI at diagnosis, and pump initiation required structured education, typically ≥3 months later. At year 2, pump use was similar between females and males, suggesting that treatment modality is unlikely to explain the observed sex differences in BMIp or LDL-C trajectories. Nonetheless, detailed longitudinal data on insulin doses and timing of pump initiation were limited, which we acknowledge as a study limitation. Our findings were consistent when using BMIzs instead of percentiles. While the sex-by-time interaction was borderline significant, the direction and magnitude of effects matched the BMIp models. This attenuation likely reflects differences in scaling, as *z*-scores compress extremes where many participants resided. Overall, the interpretation remains unchanged—females with new-onset T1D had greater early adiposity gains than males. Finally, while hyperglycemia at diagnosis may transiently affect lipid levels, the persistence of higher LDL-C in females—despite comparable glycemic control and basal insulin dosing—suggests that additional sex-specific mechanisms may be contributing to this risk.

In conclusion, our findings highlight the emergence of sex differences in BMIp and LDL-C during the early course of T1D. These differences appear independent of baseline glycemic status, insulin dose, or DKA, and may reflect underlying biological and behavioral mechanisms that begin influencing cardiometabolic health before overt puberty. Future studies should investigate the mechanistic drivers of these early disparities and explore whether sex-specific interventions—whether behavioral, pharmacologic, or both—can alter long-term cardiovascular risk. Notably, prior research suggests that females with T1D may be less responsive to lifestyle-based weight interventions than males [32], highlighting the need for tailored approaches. Our findings support sex-specific guidelines for metabolic monitoring and possibly early intervention.

## Figures and Tables

**Table 1:** Baseline (diagnosis) and follow-up (year 2) characteristics by sex.

Variable	Diagnosis		Year 2	
	Female	Male	Female	Male
Age (year)^∗∗, ∗∗∗^	9.9 ± 3.9	10.9 ± 3.9	12 ± 4	13.9 ± 3.8
HbA1c (mmol/mol)^**^	112 ± 29	104 ± 27	62 ± 19	61 ± 18
HbA1c (%)^**^	12.4 ± 2.7	11.7 ± 2.5	7.8 ± 1.7	7.7 ± 1.6
Triglycerides (mmol/L)	1.03 (0.76, 1.30)	0.96 (0.70, 1.24)	0.93 (0.64, 1.35)	0.89 (0.57, 1.26)
Total cholesterol (mmol/L)^∗, ∗∗∗^	4.07 ± 1.01	3.86 ± 0.85	4.22 ± 0.80	4.01 ± 0.80
HDL-C (mmol/L)	1.09 ± 0.28	1.11 ± 0.34	1.55 ± 0.34	1.50 ± 0.44
LDL-C (mmol/L)^∗∗, ∗∗∗^	2.51 ± 0.80	2.31 ± 0.70	2.25 ± 0.60	2.12 ± 0.65
Weight (kg)^∗∗, ∗∗∗^	31 (21, 48)	42 (26, 58)	48 (32, 62)	57 (36, 73)
BMI (kg/m^2^)^**^	16.6 (14.9, 20.7)	18.2 (15.5, 22.3)	20.4 (17.2, 24.3)	20.6 (17.6, 25)
BMI Percentile^***^	50.9 (19.2, 84.1)	63 (17.8, 93)	78.3 (56, 93.5)	76.1 (46.8, 93.8)
BMI *z*-scores	0.15 (−0.78, 1.02)	0.33 (−0.91, 1.47)	0.79 (0.15, 1.51)	0.71 (−0.08, 1.54)
Basal insulin per kg (units/kg/day)	0.28 ± 0.12	0.29 ± 0.10	0.31 ± 0.14	0.29 ± 0.14

*Note:* Values are presented as mean ± standard deviation or median (Q1, Q3) for continuous variables or percentages for categorical variables. *p*-Values derived from independent samples *t*-tests, Mann–Whitney *U* tests, or chi-square tests. Triglyceride, total cholesterol, HDL-C, and LDL-C were available for *n* = 200 females and *n* = 226 males at year 2. To convert lipid values from SI units (mmol/L) to conventional units (mg/dL), use the following factors: triglycerides: multiply by 88.57, total cholesterol, HDL-C, LDL-C, multiply by 38.67.

^*^ *p*  < 0.05 between sexes at baseline.

*⁣* ^*⁣* ^*∗∗*^^ *p*  < 0.01 between sexes at baseline.

*⁣* ^*⁣* ^*∗∗∗*^^ *p*  < 0.05 between sexes at year 2.

**Table 2:** Fixed effects from linear mixed-effects model predicting BMIp.

Parameter	Estimate	95% CI	*p*-Value
Fixed effects			
Intercept	64.55	52.46, 76.63	<0.001
Time (ref: follow-up)	−19.90	−23.08, –16.77	<0.001
Sex (ref: female)	−5.40	−10.34, –0.46	0.032
Time × sex	7.71	3.34, 12.07	<0.001
Covariates			
Age at diagnosis (years)	1.05	0.41, 1.69	0.001
HbA1c at diagnosis (%)	−0.36	−1.34, 0.62	0.471
DKA at diagnosis (yes vs. no)	2.37	−2.58, 7.31	0.347

*Note:* The model includes random intercepts for subjects and repeated BMIp measurements at diagnosis and follow-up. HbA1c was modeled in conventional units (%) for interpretability.

Abbreviations: BMIp, body mass index percentile; CI, confidence interval.

**Table 3:** Fixed effects from linear mixed-effects model predicting LDL-C.

Parameter	Estimate	95% CI	*p*-Value
Fixed effects			
Intercept	52.70	42.06, 63.34	<0.001
Time (ref: follow-up)	9.61	5.92, 13.30	<0.001
Sex (ref: female)	−5.00	−9.55, –0.44	0.032
Sex × time	−2.63	−7.68, 2.43	0.307
Covariates			
Age at diagnosis (years)	1.04	0.52, 1.56	<0.001
HbA1c at diagnosis (%)	1.66	0.86, 2.46	<0.001
Weight status (baseline)	2.54	0.37, 4.72	0.022
DKA at diagnosis (yes vs. no)	0.71	−3.26, 4.68	0.725

*Note:* Model includes random intercepts for subjects and repeated LDL-C measurements at diagnosis and 2-year follow-up. Weight status determined by BMI percentile classification at diagnosis. HbA1c was modeled in conventional units (%) for interpretability.

Abbreviations: CI, confidence interval; LDL-C, low density lipoprotein cholesterol.

## Data Availability

The data that support the findings of this study are available from the corresponding author upon reasonable request.

## Supporting Information

Supporting Information Table S1. Comparison of baseline characteristics by BMIp follow-up status. Values are presented as mean ± standard deviation for normally distributed variables, median (Q1–Q3) for skewed continuous variables, and percentages for categorical variables. *p*-Values were calculated using independent samples *t*-tests, Mann–Whitney U tests, or chi-square tests, as appropriate. HbA1c is shown in both SI units (mmol/mol) and conventional units (%). LDL-C is presented in SI units (mmol/L). Weight categories were defined using BMI percentile at diagnosis: underweight (<5th percentile), normal weight (5th–84th percentile), overweight (85th–94th percentile), and obese (≥95th percentile). Table S2. Comparison of baseline characteristics by LDL-C follow-up availability. Values are presented as mean ± standard deviation for normally distributed variables, median (Q1–Q3) for skewed continuous variables, and percentages for categorical variables. *p*-Values were calculated using independent samples *t*-tests, Mann–Whitney U tests, or chi-square tests, as appropriate. HbA1c is shown in both SI units (mmol/mol) and conventional units (%). LDL-C is presented in SI units (mmol/L). Weight categories were defined using BMI percentile at diagnosis: underweight (<5th percentile), normal weight (5th–84th percentile), overweight (85th–94th percentile), and obese (≥95th percentile). Table S3. Sensitivity analyses of BMI percentile (BMIp) trajectories using alternative age-based definitions of pubertal status. All models adjust for HbA1c and DKA status at diagnosis, sex, time (diagnosis vs. follow-up), and include a sex × time interaction. BMIp = BMI percentile for age and sex. Positive *β* values indicate higher BMIp in pubertal participants or a greater sex-related difference in trajectory. Table S4. Sensitivity analyses of BMI *z*-score (BMIz) trajectories. All models adjust for age at diagnosis, HbA1c, and DKA status at diagnosis, and include sex, time (diagnosis vs. follow-up), and a sex × time interaction. BMIz = BMI *z*-score for age and sex. Positive *β* values indicate higher BMIz in the specified group or a greater sex-related difference in trajectory. Table S5. Sensitivity analyses of LDL-C trajectories using alternative age-based definitions of pubertal status. All models adjust HbA1c, DKA status, and weight status at diagnosis, sex, time (diagnosis vs. follow-up), and include a sex × time interaction. LDL-C values are in mg/dL. The puberty variable compares pubertal vs. prepubertal groups; positive *β* indicates higher LDL-C in pubertal participants. Weight status at diagnosis was based on BMI percentile for age and sex.
