# Supplementary material for: Sex-Based Differences in BMI and LDL-C Trajectories Following Type 1 Diabetes Diagnosis in Youth
Source: Pediatr Diabetes. 2025 Nov 11;2025:7996152. doi: 10.1155/pedi/7996152 (PMC12626691; doi:10.1155/pedi/7996152)
Supplement: Supporting Information — Table S1. Comparison of baseline characteristics by BMIp follow-up status. Values are presented as mean ± standard deviation for normally distributed variables, median (Q1–Q3) for skewed continuous variables, and percentages for categorical variables. p-Values were calculated using independent samples t-tests, Mann–Whitney U tests, or chi-square tests, as appropriate. HbA1c is shown in both SI units (mmol/mol) and conventional units (%). LDL-C is presented in SI units (mmol/L). Weight categories were defined using BMI percentile at diagnosis: underweight (<5th percentile), normal weight (5th–84th percentile), overweight (85th–94th percentile), and obese (≥95th percentile). Table S2. Comparison of baseline characteristics by LDL-C follow-up availability. Values are presented as mean ± standard deviation for normally distributed variables, median (Q1–Q3) for skewed continuous variables, and percentages for categorical variables. p-Values were calculated using independent samples t-tests, Mann–Whitney U tests, or chi-square tests, as appropriate. HbA1c is shown in both SI units (mmol/mol) and conventional units (%). LDL-C is presented in SI units (mmol/L). Weight categories were defined using BMI percentile at diagnosis: underweight (<5th percentile), normal weight (5th–84th percentile), overweight (85th–94th percentile), and obese (≥95th percentile). Table S3. Sensitivity analyses of BMI percentile (BMIp) trajectories using alternative age-based definitions of pubertal status. All models adjust for HbA1c and DKA status at diagnosis, sex, time (diagnosis vs. follow-up), and include a sex × time interaction. BMIp = BMI percentile for age and sex. Positive β values indicate higher BMIp in pubertal participants or a greater sex-related difference in trajectory. Table S4. Sensitivity analyses of BMI z-score (BMIz) trajectories. All models adjust for age at diagnosis, HbA1c, and DKA status at diagnosis, and include sex, time (diagnosis vs. follow-up), and a sex × time inter [file 7996152.f1.docx]

# **Supplementary Table 1.** Comparison of Baseline Characteristics by BMIp Follow-Up Status

| Variable | With Follow-Up Data | Without Follow-Up Data | p-value |
| --- | --- | --- | --- |
| N | 476 | 76 |  |
| Age (years) | 10.3 ± 3.8 | 11.5 ± 4.0 | 0.08 |
| HbA1c (mmol/mol) | 108 ± 28 | 112 ± 30 | 0.08 |
| HbA1c (%) | 12.1 ± 2.6 | 12.4 ± 2.7 | 0.48 |
| LDL-C (mmol/L) | 2.37 ± 0.74 | 2.50 ± 0.78 | 0.30 |
| BMIp (%) | 56.2 [20.5–89.3] | 58.1 [24.0–89.7] | 0.77 |
| Sex (%, female) | 47.2% | 31.0% | 0.04 |
| DKA at diagnosis (%) | 48.0% | 55.0% | 0.39 |
| Weight Category at diagnosis | Underweight: 10.8%  Normal weight: 59.8%  Overweight: 11.4%  Obese: 18.0% | Underweight: 19.0%  Normal weight: 40.5%  Overweight: 9.5%  Obese: 31.0% | 0.04 |

**Supplementary Table 2.** Comparison of Baseline Characteristics by LDL-C Follow-up Availability

| Variable | Follow-up Available | Follow-up Missing | p-value |
| --- | --- | --- | --- |
| N | 426 | 116 |  |
| Age (years) | 10.40 ± 3.83 | 10.44 ± 3.83 | 0.98 |
| HbA1c (mmol/mol) | 108 ± 28 | 106 ± 28 | 0.73 |
| HbA1c (%) | 12.10 ± 2.58 | 11.97 ± 2.60 | 0.73 |
| LDL-C (mmol/L) | 2.31 ± 0.71 | 2.38 ± 0.75 | 0.54 |
| BMIp (%) | 63.6 [25.2–91.7] | 71.6 [28.5–96.5] | 0.11 |
| Sex (female) | 47.1% | 48.2% | 0.87 |
| DKA at diagnosis | 48.6% | 42.6% | 0.43 |
| Weight category at diagnosis | Underweight: 11.3%  Normal weight: 59.0%  Overweight: 11.9%  Obese: 17.8% | Underweight: 7.1%  Normal weight: 66.1%  Overweight: 7.1%  Obese: 19.6% | 0.51 |

# **Supplementary Table 3**. Sensitivity Analyses of BMI Percentile (BMIp) Trajectories Using Alternative Age-Based Definitions of Pubertal Status

| Model | Prepubertal Definition | Puberty Effect (β) (95% CI) | p-value (Puberty) | Sex × Time Interaction (β) (95% CI) | p-value (Interaction) |
| --- | --- | --- | --- | --- | --- |
| Model 1 | Females <9 yrs, Males <10 yrs | 6.67 (1.60, 11.74) | 0.010 | 7.72 (3.36, 12.08) | <0.001 |
| Model 2 | Females <9 yrs, Males <11 yrs | 6.77 (1.16, 11.73) | 0.008 | 7.73 (3.36, 12.09) | <0.001 |
| Model 3 | Females <10 yrs, Males <11 yrs | 8.29 (3.41, 13.17) | <0.001 | 7.72 (3.36, 12.08) | <0.001 |

| **Supplementary Table 4.** Fixed Effects from Linear Mixed-Effects Model Predicting BMIz | | | |
| --- | --- | --- | --- |
| **Parameter** | **Estimate** | **95% CI** | **p-value** |
| **Fixed Effects** |  |  |  |
| Intercept | 0.70 | –7.84, 9.23 | 1.000 |
| Time (ref: follow-up) | –0.42 | –0.64, –0.20 | <0.001 |
| Sex (ref: female) | 0.21 | –0.03, 0.40 | 0.086 |
| Time × Sex | –0.32 | –0.64, 0.01 | **0.055** |
| **Covariates** |  |  |  |
| Age at diagnosis (years) | 0.04 | 0.02, 0.06 | <0.001 |
| HbA1c at diagnosis (%) | –0.05 | –0.08, –0.02 | 0.002 |
| DKA at diagnosis (yes vs. no) | 0.10 | –0.06, 0.26 | 0.205 |

# **Supplementary Table 5.** Sensitivity Analyses of LDL-C Trajectories Using Alternative Age-Based Definitions of Pubertal Status

| Model | Prepubertal Definition | Puberty Effect (β) (95% CI) | p-value (Puberty) | Weight Category Effect (β) (95% CI) | p-value (Weight) |
| --- | --- | --- | --- | --- | --- |
| Model 1 | Females <9 yrs, Males <10 yrs | 6.50 (2.40, 10.60) | 0.002 | 2.79 (0.61, 4.96) | 0.012 |
| Model 2 | Females <9 yrs, Males <11 yrs | 6.54 (2.52, 10.56) | 0.001 | 2.81 (0.64, 4.99) | 0.011 |
| Model 3 | Females <10 yrs, Males <11 yrs | 6.84 (2.88, 10.81) | <0.001 | 2.76 (0.59, 4.94) | 0.013 |
